# Unveiling Rare Breast Neoplasms: Diagnostic Challenges and Insights

**DOI:** 10.15190/d.2024.19

**Published:** 2024-12-31

**Authors:** Rasheeda Mohamedali, Nilay Nishith, Rahul Raj, Nishtha Ahuja, Aishwarya Sharma, Puneet Kaur Somal, Ravikiran N Pawar, Sankalp Sancheti

**Affiliations:** ^1^Department of Pathology, Homi Bhabha Cancer Hospital and Research Centre, Tata Memorial Centre, Homi Bhabha National Institute (HBNI), New Chandigarh, Punjab, India

**Keywords:** Breast, diagnosis, neoplasm, oncology, unusual.

## Abstract

Breast neoplasm encompasses a diversified range of different diseases characterized by unique biological and pathological features, clinical presentation, response to treatments, clinical behavior, and outcome. The histological variability has profound prognostic implications, thus playing a pivotal role in diagnosing breast neoplasm. Special histologies can occur rarely, and most information on outcomes and treatments is mainly derived from small series and case reports. Thus, reporting such unusual occurrences is of utmost importance. This is a retrospective study of four years in which 11 cases of breast neoplasm diagnosed on histopathology were assessed. The clinical details were acquired from an electronic search. The hematoxylin and eosin and immunohistochemistry slides were retrieved from the archives to review the histopathological features. The patient’s age ranged from 32 to 72 years (median: 51 years).  Eleven unusual cases of breast neoplasms were diagnosed and categorized according to the latest World Health Organisation (WHO) classification. These cases include rare and salivary gland type tumors (2 cases), neuroendocrine neoplasms (1 case), hamartomas of the breast (2 cases), fibroblastic and myofibroblastic tumors of the breast (1 case), peripheral nerve sheath tumor (1 case) and hematolymphoid tumors of the breast (4 cases). The management of unusual breast cancer histotypes represents a real challenge in daily clinical practice. Our data suggest that these variants are distinct clinicopathological entities with unique hormonal receptor statuses. The occurrence of such entities is rare, and they demonstrate different clinical behaviors and responses to treatment, suggesting that a few genomic-specific events might also drive them. Therefore, it is important to indulge in future research on such rare breast neoplasms.

## Introduction

Breast cancer is the most common malignancy in women and is associated with significant mortality and morbidity. Tumors of the breast impact approximately 2.1 million women each year and cause the greatest number of cancer-related deaths among women^1^. There has been a dramatic increase in breast cancer cases worldwide, and thus, this arena represents a top biomedical research priority. These cancer rates are expected to be disproportionately high in developing countries^2^.In women aged less than 45 years, breast cancer is the leading cause of cancer-related deaths, as the cancers tend to be more aggressive in the younger age group.

The spectrum of breast neoplasms encompasses the most common benign neoplasm, fibroadenoma, to the most common malignant neoplasm, invasive carcinoma of no special type (ductal) (IDC-NST). Other than these entities, there is a wide variety of tumors that occur rarely but are included in the World Health Organization (WHO) classification^3^. These unusual tumors often display a diversified biological behavior and have challenging modes of therapeutic and prognostic information.

Although rare breast neoplasms are increasingly being recognized globally, there remains a significant gap in data from underrepresented populations. Most published studies are limited to Western cohorts and focus predominantly on individual subtypes or case series. This study is unique in systematically analyzing rare breast neoplasms in an Indian cohort, applying the updated WHO classification, and bridging the gap between diagnostic challenges and clinical outcomes. By addressing histopathological and immunohistochemical features, this work provides a valuable diagnostic framework tailored to the regional population and highlights clinical implications in management.

With this background, the aim of our study is to present our institutional experience on unusually encountered entities and the diagnostic challenges faced during their clinical, histopathological, and therapeutic evaluation.

## Materials and Methods

This was a retrospective study conducted in the Department of Pathology from March 2021 to March 2024. Out of 3841 cases of breast neoplasms, 11 cases of rare neoplasms were identified by an initial electronic search and through departmental records. The clinical and radiological details were compiled. Histological slides were reviewed by two independent pathologists and correlated with their immunohistochemistry (IHC), and the characteristic lesions were analyzed and classified according to the latest WHO classification of breast tumors ^3^. Since it is a retrospective study, no additional intervention or invasive procedure was performed on any patient, precluding any ethical violation.

## Results

A total of 3841 cases of breast neoplasms were reported in the study period. Out of these cases, the most common cases were IDC-NST (65% of the total cases) in the malignant series and fibroadenoma (2% of the total cases) in the benign. All the cases elucidated were evaluated under the categories of the WHO Blue Book, and the unusual ones were selected: Rare and salivary gland type tumors (2 cases), neuroendocrine neoplasms (1 case), hamartomas of the breast (2 cases), fibroblastic and myofibroblastic tumors of the breast (1 case), peripheral nerve sheath tumor (1 case) and hematolymphoid tumors of the breast (4 cases).

The patient’s age ranged from 32 years to 72 years (median: 51 years). A summary of these rare histological subtypes, including their clinical presentation, histopathological and IHC findings, treatment and outcome are presented in Table 1.

**Table 1:** Comprehensive Clinicopathological, Treatment, and Outcome Profiles of Rare Breast Neoplasms

Case No.	Age (yrs)	Duration of Illness (months)	BIRADS Score	Histopathological Diagnosis	Immunohistochemical Markers	Treatment Modalities	Clinical Outcomes
1	54	12	4a	Adenoid cystic carcinoma	Positive: GATA3, CK7, CD117, CK5/6, p63; Negative: ER, PR, HER2	Lumpectomy + adjuvant radiotherapy	Disease-free at 17 months
2	62	36	4c	Adenoid cystic carcinoma	Positive: CK7, CD117, p63; Negative: ER, PR, HER2	BCS + adjuvant radiotherapy	Disease-free at 12 months
3	49	7	4c	Mixed ductal neuroendocrine carcinoma	Positive: AE1/AE3, p53, synaptophysin, CD56, high Ki67 index; Negative: ER, PR, HER2	MRM + chemoradiation	Disease-free at 24 months
4	32	Incidental detection (screening for lung lesion)	3	Mammary hamartoma	Nil	Lumpectomy	Follow-up data is not available
5	40	120	3	Mammary hamartoma	Nil	Observation	Follow-up data is not available
6	61	120	3	Myofibroblastoma	Positive: ER, PR, AR, CD34, desmin; Negative: AE1/AE3, SMA, p63	Lumpectomy	Disease-free at 15 months
7	72	0.5	4c	Granular cell tumor	Positive: S100, SOX10, TFE3; Negative: GATA3, ER, PR, HER2	Wide local excision	Disease-free at 7 months
8	45	3	4a	Diffuse large B cell lymphoma	Positive: CD45, CD20, BCL2, weak BCL6, MUM1, high Ki67; Negative: CD3, synaptophysin, ER, PR	Chemotherapy (R-CHOP regimen)	Progression at 8 months with new lymphadenopathy
9	47	3	4a	Diffuse large B cell lymphoma	Positive: CD45, CD20, BCL2, high Ki67 index; Negative: CD3, ER, PR, HER2	Chemotherapy (R-CHOP regimen)	Lost to follow-up
10	53	3	4b	B-cell Non-Hodgkin lymphoma, low grade	Positive: CD20, PAX5, BCL2, low Ki67 index; Negative: CD3, CD5	Chemotherapy (low-intensity regimen)	Follow-up data is not available
11	53	1.5	5	Plasmacytoma	Positive: CD138; Negative: CD45, CD56	VRd regimen + Tamoxifen	Very good partial response

### Rare and salivary gland-type tumors - Case 1

#### Clinicoradiological findings

A 54-year-old female presented with a painless lump in the left breast, with an inverted nipple measuring 4 x 5 cm in size in the 9 o’clock position for 1 year. There was a scar from a previous incisional biopsy performed 4 months back from elsewhere, which was reported as IDC-NST. No lymphadenopathy was noted. Bilateral mammography showed a well-defined lobulated lesion measuring 5 x 3 cm in the inner half of the left breast at the 8 to 10 o’clock position. Positron emission tomography-computed tomography (PET-CT) showed no avid uptake in the lymph node.

#### Histopathology and IHC findings

**Figure 1:**
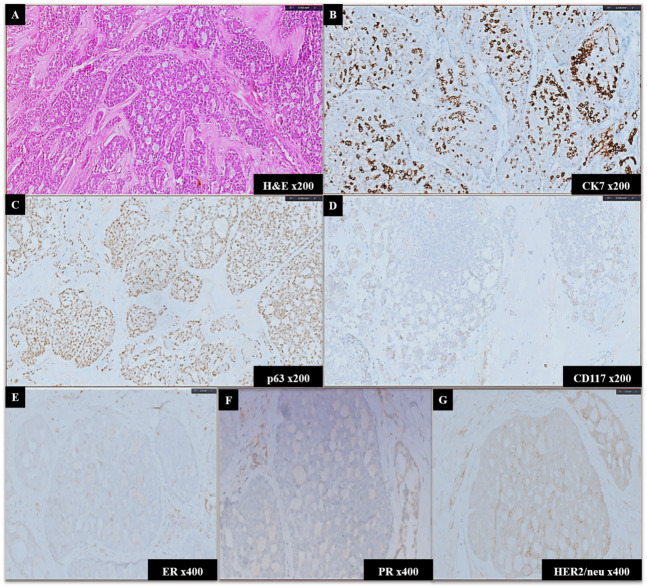
Histopathology and immunoprofile of Adenoid cystic carcinoma (A-G) A. Histopathology: The tumor cells are arranged in cribriform architecture with true glandular spaces and pseudolumina filled with stromal matrix (Hematoxylin and Eosin, x200). B-G. Immunohistochemistry (IHC): CK7 and CD117 highlight the epithelial cells; B-C. IHC, x200; D. p63 highlights the myoepithelial cells (IHC, x200); E-G. ER, PR, and HER2/neu are negative (IHC, x400).

A core needle biopsy was performed, which showed an infiltrating tumor composed of a dual population of cells (epithelial and myoepithelial). These cells were arranged in a tubular pattern with a slit-like lumen and occasionally in a cribriform pattern (Figure 1 a). Frequent mitotic figures and apoptotic bodies are seen. By immunohistochemistry, GATA-3, CD117, CK7, and CK5/6 were diffusely positive in ductal epithelial cells, which showed focal positivity for EMA. p63 and S100 highlighted the myoepithelial cells (Figure 1 b-d). The tumor cells were triple negative for estrogen receptor, progesterone receptor, and HER2/neu (Figure 1 e-g). A final diagnosis of adenoid cystic carcinoma (ACC) was rendered.

Later, a left breast lumpectomy specimen was received, which showed findings similar to those above. The margins were free of tumor, and the lymph nodes were negative for metastasis. The patient underwent radiation therapy, was on follow-up for 1.5 years, and did not have any recurrence.

### Rare and salivary gland-type tumors - Case 2

#### Clinicoradiological findings

A 62-year-old female presented with a 4 x 4 cm lump at a 3 o’clock position in the left breast for 3 years. Her mammography revealed a well-defined mass with associated architectural distortion and lobulated margins. (BIRADS 4c). A CT thorax was performed, and no significant lymphadenopathy was observed.

#### Histopathology and IHC findings

A core biopsy was performed, which showed scanty foci of invasive carcinoma composed of epithelial and myoepithelial cells arranged in cribriform architecture with true glandular spaces and pseudolumina. The glandular spaces are lined by epithelial-type cells that produce mucins; the pseudolumina are lined by the myoepithelial cells and filled with stromal matrix. By immunohistochemistry, CK7 and CD117 highlighted the epithelial cells, and p63 highlighted the myoepithelial cells. The overall features were consistent with adenoid cystic carcinoma. IHC for the prognostic and predictive biomarkers were triple negative for ER, PR, and HER2/neu. A left breast conservation surgery (BCS) was performed, which showed a single focus of a similar tumor. The patient was on follow-up for 1 year after completion of radiotherapy.

### Neuroendocrine neoplasm - Case 3

#### Clinicoradiological findings

A 49-year-old female came with complaints of an ulcer in the left breast for 7 months. On examination, it was an 8 x 7 cm lateral ulcer with palpable axillary lymphadenopathy. A contrast-enhanced CT of the thorax, abdomen, and pelvis was performed, which showed a well-defined heterogeneously enhancing necrotic and ulcerative oval lesion with macrolobulated margins that measured 10.3 x 14.7 x 13 cm. A few suspicious lymph nodes measuring 2.4 x 1 cm were also identified.

#### Histopathology and IHC findings

**Figure 2:**
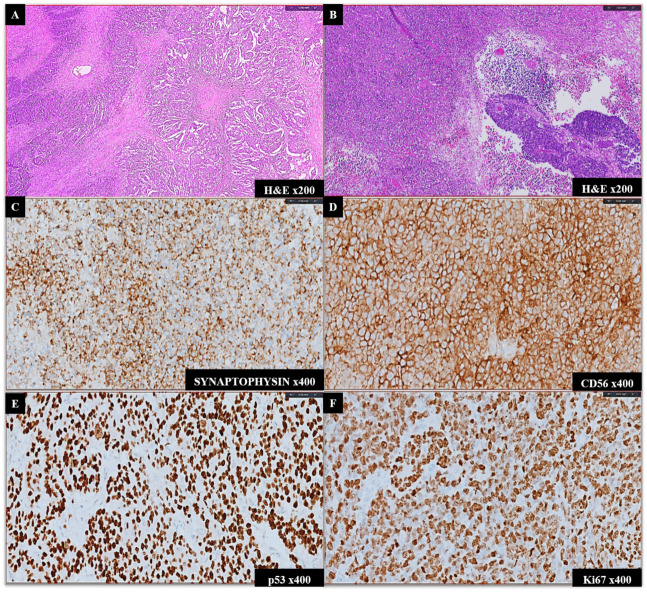
Histopathology and immunoprofile of Mixed Ductal and Neuroendocrine Carcinoma (A-F) A-B. Histopathology: A. The tumor cells are arranged in glandular pattern (right side) and in diffuse sheets (left side) with areas of necrosis in B; A-B. Hematoxylin and Eosin, x200). C-F. Immunohistochemistry (IHC): The small cell neuroendocrine carcinoma component is diffusely positive for synaptophysin, CD56, and p53; C-E. IHC, x400); F. MIB-1 labeling index is 78-80% in the highest proliferative area (IHC, x400).

Ultrasound-guided FNAC was performed from the breast lesion, which showed clusters of tumor cells with an occasional acinar arrangement and was reported as ductal carcinoma. A left modified radical mastectomy (MRM) specimen was received. On gross evaluation, the tumor showed extensive areas of necrosis and involved and ulcerated both the skin and nipple areola complex. Histopathological examination showed a mixed tumor with features of both ductal carcinoma (60%) and small cell neuroendocrine carcinoma (40%) (Figure 2 a,b). Lymphovascular emboli were noted, and the tumor showed metastatic deposits in seven out of 30 lymph nodes dissected. By IHC, the tumor cells showed patchy positive staining for AE1/AE3, while they were negative for GATA3. The small cell neuroendocrine carcinoma component was diffusely positive for synaptophysin and CD56, while they were negative for chromogranin (Figure 2 c,d). These tumor cells also showed diffuse and strong positivity for p53 (mutant-type staining) (Figure 2 e). The MIB-1 labeling index was 78-80% in the highest proliferative area. (Figure 2 f). IHC for ER, PR, and HER2/neu were all negative. The patient underwent chemoradiation and has a recurrence free interval of two years.

### Hamartomas of the breast - Case 4

#### Clinicoradiological findings

A 32-year-old female presented with backache. A well-defined 3.5 x 2.8 cm space occupying the lesion was detected in the right lung, perihilar location with small cystic necrosis. As the patient was investigated for this primary tumor, she had a new onset ill-defined left breast lump at the 12 o’clock position. Ultrasound revealed a hypoechoic oval lesion with an angulated margin measuring 1.6 x 1.25 cm. Mammography also revealed a similar tumor with a BIRADS category 3.

#### Histopathological findings

CT-guided FNA was performed from the lung mass, which showed loosely cohesive clusters and singly scattered tumor cells with evidence of molding and was reported as neuroendocrine carcinoma. However, the core biopsy section revealed epithelioid cells with a perivascular arrangement. A pneumonectomy was done, a large panel of IHC was performed, and the tumor was reported as sclerosing hemangioma.

**Figure 3:**
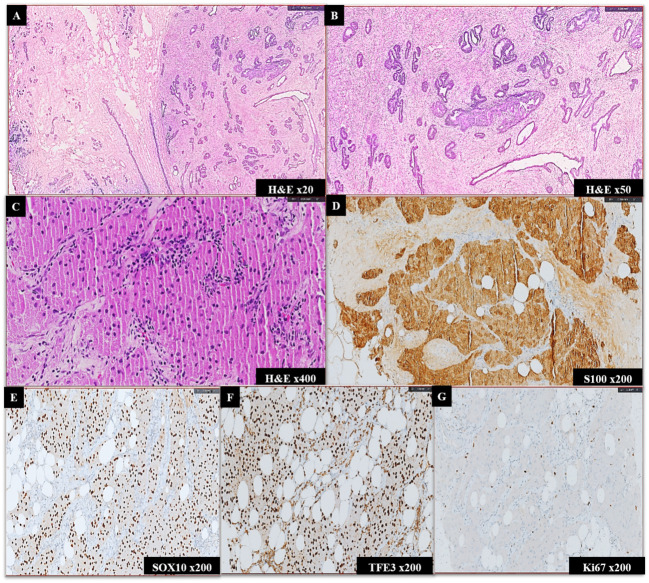
Histopathology of Mammary Hamartoma (A-B) and histopathology and immunoprofile of Granular Cell Tumor (C-G) A-B. The section shows a lesion comprising mammary glandular tissue, fibrous stroma, and adipose tissue (Hematoxylin and Eosin, x20 and x50). C. Histopathology: Sheets and nests of neoplastic, polygonal, granular cells embedded in a collagenous stroma (Hematoxylin and Eosin, x400). D-G. Immunohistochemistry (IHC): The neoplastic cells are positive for S100, SOX10, and TFE3; D-F.: IHC, x400). MIB1 labeling index: 2-3% in the highest proliferating area (IHC, x400).

A left breast lumpectomy was also performed, suspecting metastasis, which grossly did not reveal a definite tumor. On microscopic examination, a hamartomatous lesion comprising mammary glandular tissue, fibrous stroma, and adipose tissue was found (Figure 3 a,b).

### Hamartomas of the breast - Case 5

#### Clinicoradiological findings

A 40-year-old female came with a lump in her left breast for 10 years associated with cyclical pain. Mammography showed a 2.9 x 1.7 cm sized isoechoic mass at 6-8 o’clock position with ACR BIRADS category 3.

#### Histopathology and IHC findings

A core biopsy was performed, and the section showed a benign breast lesion comprising ducts, lobules, and scattered adipose cells, along with prominent intralobular fibrosis. A diagnosis of mammary hamartoma was rendered.

### Fibroblastic and myofibroblastic tumors - Case 6

#### Clinicoradiological findings

A 61-year-old female presented with a lump in her right breast for 10 years. On examination, a 5 x 5 cm mass was identified in the upper inner quadrant with a palpable right axillary lymphadenopathy. Mammography revealed a large, well-encapsulated heterogenous lesion with a size of 8.4 x 6.6 cm. As there was no architectural distortion or calcification, the findings favored a hamartomatous lesion.

**Figure 4:**
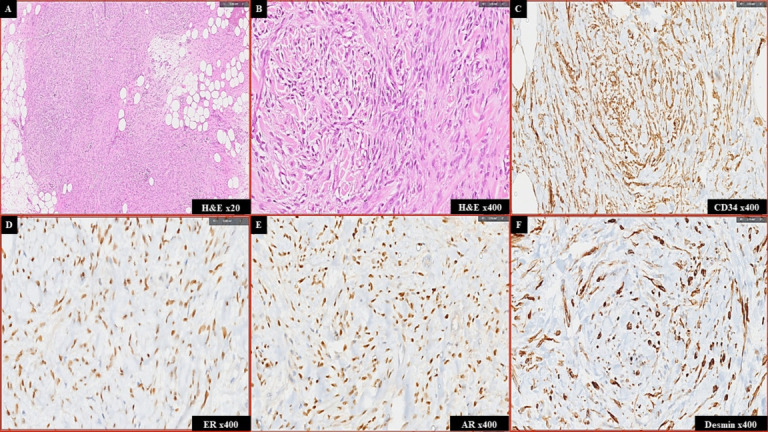
Histopathology and immunoprofile of Myofibroblastoma (A-F) A-B. Histopathology: Presence of short fascicles of the spindle to plump cells with intervening keloid-like collagen fibers and a sprinkling of mast cells (Hematoxylin and Eosin, x200 and x400). C-F. Immunohistochemistry (IHC): The neoplastic cells were positive for CD34, ER, AR and desmin (IHC, x400).

#### Histopathology and IHC findings

The biopsy section showed no features of hamartoma or malignancy. FNAC of the lymph node was reported as reactive lymphadenitis. Following this, a lumpectomy was received, which, on gross examination, showed a well-defined grey-white to yellowish lesion. The lesion on microscopy showed benign mesenchymal neoplasm with vague nodular cellular areas admixed with fat. The cellular areas exhibited short fascicles of the spindle to plump cells with intervening keloid-like collagen fibers and a sprinkling of mast cells (Figure 4 a-b). An IHC panel was done to delineate the tumor. These spindle cells were positive for ER, PR, AR, CD34, and desmin (Figure 4 c-f), while negative for AE1/AE3, SMA, and p63. A diagnosis of myofibroblastoma was rendered, and the patient was on follow-up for 15 months with no recurrence.

### Peripheral nerve sheath tumor - Case 7

#### Clinicoradiological findings

A 72-year-old female presented with the complaint of a lump in the left breast for 15 days. Her clinicopathological examination revealed an irregular, partly circumscribed, and partly spiculated dense mass in the upper quadrant measuring 2.5 x 2.3 cm. A BIRADS score of 4c was rendered.

#### Histopathology and IHC findings

A core needle biopsy was performed, which showed sheets and nests of neoplastic cells embedded in a collagenous stroma. These cells were round to polygonal in shape with indistinct cell borders, abundant eosinophilic granular cytoplasm, round to oval nuclei, and small conspicuous nucleoli. (Figure 3 c) On IHC, the neoplastic cells were positive for S100, SOX10, and TFE3 (Figure 3 d-f), while they were negative for GATA3, ER, PR, HMB45, and HER2/neu. MIB 1 labeling index: 2-3% in the highest proliferating area (Figure 3 g). A final diagnosis of granular cell tumor (GCT) was given. A lumpectomy was performed, which showed a similar tumor. The patient was on follow-up for 7 months with no recurrence.

### Hematolymphoid tumors of the breast - Cases 8 and 9

#### Clinicoradiological findings

Two patients (a 45-year-old and a 47-year-old) presented on different occasions with similar complaints of a right breast lump for 3 months. Both patients underwent a core needle biopsy following a radiological BIRADS score of 4a for the lesion.

#### Histopathology and IHC findings

**Figure 5:**
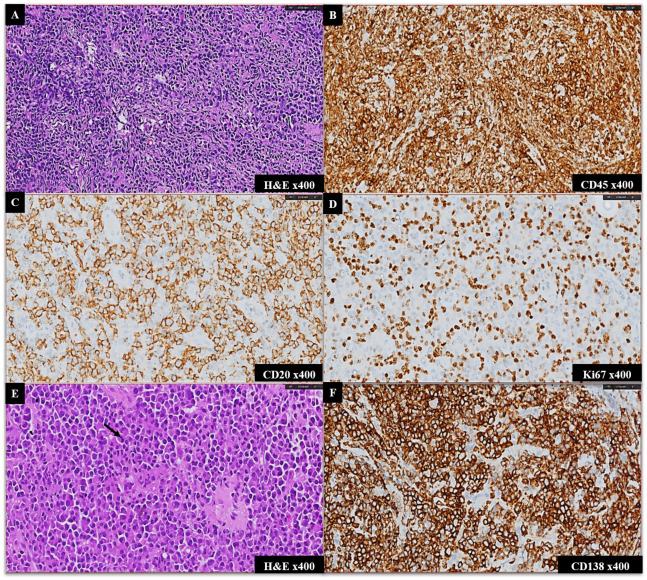
Histopathology and immunoprofile of Diffuse large B cell Non-Hodgkin lymphoma (A-D) and Plasmacytoma (E-F) A. Histopathology: High-grade malignant tumor composed of atypical large cells (A. Hematoxylin and Eosin, x400). B-D. Immunohistochemistry (IHC): These atypical large cells are diffusely positive for CD45 and CD20 (B-C. IHC, x400). MIB1 labeling index is 68-70% in the highest proliferating areas. (D. IHC, x400). E-F. Plasmacytoma; E. Histopathology: Sheets of plasma cells with occasional mitosis (black arrow) (Hematoxylin and Eosin, x400). F. Immunohistochemistry (IHC): The neoplastic plasma cells were positive for CD138 (IHC, x400).

The histological and IHC profiles were similar. The section showed a high-grade malignant tumor composed of atypical large cells. (Figure 5 a) By immunohistochemistry, these atypical large cells are diffusely positive for CD45, CD20, and BCL2, weakly positive for BCL6, and MUM1, while they are negative for CD34, AE1/AE3, CK5/6, ER, PR, and HER2/neu, Vimentin, CD56, CD99, and synaptophysin. CD3 highlights the background T cells. (Figure 5 b,c) The MIB1 labeling index is 68-70% in the highest proliferating areas. (Figure 5 d) The features were suggestive of diffuse large B-cell non-Hodgkin lymphoma. A PET-CT was performed, which did not reveal any lesion elsewhere. A cerebrospinal fluid and bone marrow examination was also done, and the results were within normal limits. Both patients were provided with chemotherapy. One of the patients was lost to follow-up, and the other presented with disease progression after 8 months. The breast mass recurred, and she had multiple axillary, abdominal, and inguinal lymph nodes. The patient was not willing to undergo intensive palliative chemotherapy.

### Low-grade non-Hodgkin B - Case 10

#### Clinicoradiological findings

A 53-year-old female presented with a left breast lump measuring 1.2 x 1.1cm. Mammography revealed a hyperdense mass with micro-lobulated margins, BIRADS 4b. On routine examination, her total leucocyte count was 33 x 10^9^/L with absolute lymphocytosis. A detailed history was taken, and the patient conveyed to have leukocytosis for 4 years. There were no B symptoms elucidated. During flow cytometric analysis, the atypical lymphocytes were CD5+, CD200+, CD23-, and ROR1-.

#### Histopathology and IHC findings

A biopsy was taken from the breast nodule, which showed atypical lymphoid proliferation comprising of small to medium-sized cells. By immunohistochemistry, these lymphoid cells are positive for CD20, PAX5, and BCL2, while they are negative for BCL6. CD3 and CD5 highlight background T cells, while CD23 highlights the occasional dendritic cell networks. MIB1 labeling index is approximately 25% in the highest proliferating areas. Hence, a diagnosis of low-grade non-Hodgkin B cell lymphoma was rendered.

### Plasmacytoma of the breast - Case 11

#### Clinicoradiological findings

A 53-year-old woman presented with a right breast lump, present for 1.5 months, measuring 4 x 3 cm. A lumpectomy had been performed elsewhere, as the mass was classified as BIRADS 5 on mammography, with a suspicious ipsilateral axillary lymph node. Initial hematological and serological tests at our institute indicated mild anemia and a reversal of the albumin-globulin ratio.

#### Histopathology and IHC findings

The lumpectomy specimen sections were reviewed at our institute, revealing histomorphological features consistent with invasive breast carcinoma of no special type (ductal) (IDC-NST) grade 3. The tumor was positive for ER and PR, but negative for HER2/neu. One month later, the patient presented with a recurrence in the contralateral breast, confirmed by mammography. The mammogram of the left breast showed an irregular, high-density mass in the upper outer quadrant, measuring 2.2 x 2.8 x 2.5 cm, with pleomorphic calcifications within the mass and grouped calcifications. A subsequent PET-CT scan revealed nodular right pleural thickening and lytic lesions in the sternum and vertebrae (D2, D7-L5). A core biopsy of the left breast mass demonstrated aggregates of plasma cells, which were positive for CD138 and negative for CD45 and CD56, with no evidence of invasive or in-situ carcinoma. Bone marrow aspirate and biopsy showed no excess plasma cells. Serum protein electrophoresis revealed a band in the M region, which was kappa-restricted. Based on these findings, the patient was diagnosed with plasmacytoma of the breast and multiple myeloma. She has been started on a VRd regimen for multiple myeloma alongside tamoxifen for her breast cancer. After four cycles of chemotherapy, she has shown a very good partial response.

## Discussion

Breast neoplasms are the most commonly reported cause of morbidity and mortality among women globally, with rare cases reported in males^4^. The scenario in developing countries is more profound due to a lack of awareness of screening programs and practices. In the WHO classification, there are more than a dozen subtypes that occur rarely but still are well elaborated^3^. They account for less than 10% of breast tumors and exhibit diversified biological behaviors^5^. Hence, it is critical to understand their major characteristics to make the best treatment decision and predict prognosis.

In our study, we have shed light on the various rare breast neoplasms encountered during our routine practice. Regarding unusual breast lesions, we found 2 cases in the benign category and 11 unusual cases in the malignant category. The histomorphological and IHC of rare breast neoplasms have been highlighted only in a handful of Indian studies ^4-6^. Hence, we present a brief review of these rarely occurring breast neoplasms.

### Adenoid cystic carcinoma

ACC of the breast is an extremely rare tumor, accounting for <0.1% of all breast cancers diagnosed ^7^. Various studies have reported the occurrence of ACC in the breast and have evaluated the morphological and immunohistochemical features ^7-10^.

Clinically, the patient presents with a circumscribed, painful lump. Histologically, the diagnostic criteria for ACC is the presence of a biphasic cellular pattern comprised of myoepithelial and epithelial cells^8^. ACC is a triple-negative breast carcinoma (TNBC) but has a favorable prognosis in contrast to other TNBCs. They rarely metastasize and have a 10-year survival rate of 85-100% ^9^.

Due to their rare occurrence and close resemblance to invasive breast carcinomas, ACC could be misdiagnosed as IDC-NST on core biopsies. The differentials to be aware of when diagnosing an ACC are the benign entity collagenous spherulosis (CS) and the malignant entity cribriform carcinoma ^10^.

CS is characterized by the presence of multiple intraluminal, rounded clusters (‘spherules’) of eosinophilic, collagenous, fibrillar material, which are surrounded by a bland proliferation of epithelial and myoepithelial cells. In CS, the presence of ductal epithelium in cellular lobules and the formation of lumina of varying sizes makes it much easier to assess ^11^. The absence of CD117 staining in CS, which highlights the epithelial cells in ACC, is yet another aid. The cribriform carcinoma is morphologically difficult to differentiate from ACC and warrants a panel of IHC, of which ER and PR are usually positive ^12^.

### Neuroendocrine carcinoma

Neuroendocrine carcinoma of the breast is a rare tumor with an incidence reported to range from less than 1-5% of breast cancers ^13^. These are hypothesized to arise from neuroendocrine cells, which are constitutively found in the breast. They belong to the luminal subtype and are usually HER2/neu negative.

In a retrospective investigation by Bogina et al., out of 55 early neuroendocrine breast carcinoma, disease-free survival was significantly worse when compared with non-neuroendocrine carcinoma^14^. Their therapeutic modality depends upon the biological stage of the disease and the risk of recurrence. Adjuvant chemoradiation and hormonal therapy are the mainstays in the treatment.

### Mammary hamartoma

Hamartomas are benign masses of variable size, originating from the normal cellular components of tissue but in a disorganized and haphazard manner. Mammary hamartoma is estimated to account for about 4.8% of benign breast lesions ^14^. They usually present as painless lumps, often mimicking fibroadenomas clinically.

In this growing era of employing core biopsies, the difficulty of diagnosing mammay hamartoma has been exponentially higher. The pathologists usually find fibrous fatty stroma, including various amounts of epithelial elements that look identical to a usual breast parenchyma, causing its misdiagnosis. This entity can be differentiated from a fibroadenoma by looking for adipose tissue components, which are present in mammary hamartomas. This entity may be associated with Cowden syndrome, making their recognition of paramount importance. A surgical excision with clear margins suffices for its treatment ^15^.

### Myofibroblastoma

Myofibroblastoma is a slow-growing, well-circumscribed lobulated neoplasm of the breast arising from the precursor cells of the mammary stroma. The prevalence is unknown, with an estimated occurrence of <1% of all breast neoplasms ^16^. This entity was originally described in elderly males with gynecomastia. Other sites precluding its presence include inguinal, perianal, trunk, lower limbs, and intra-abdominal ^17^.

Myofibroblastoma shares close similarities with solitary fibrous tumors with the presence of spindle cells arranged in short fascicles with interspersed keloid-like fibers. A consistent CD34 and Vimentin expression with a negative STAT6 differentiates it from a solitary fibrous tumor ^18^. Another differential is a low-grade fibromatosis-like metaplastic carcinoma, which may be ruled out by negative CD34 immunostaining ^19,20^. Surgical excision is the treatment of choice, as these tumors show a low recurrence rate.

### Granular cell tumor

GCTs are estimated to account for 6-8% of cases ^3^. Although they commonly occur in the tongue, it is a known fact that they can develop anywhere in the body. The lesional cells presumably have Schwann cell origin, and thus, this tumor expresses S100. A study by Swamy et al. proposed that GCTs of the breast can arise as a synchronous lesion with GCTs elsewhere in the body or as a metachronous lesion ^21^.

Although it is much easier to recognize in histology, ultrasonography may reveal irregular hypoechoic lesions with posterior acoustic shadowing, indicating cancer. Less than one percent of mammary GCTs are cancerous ^22^. Malignant lesions are those that are more than 5 cm in size and have enhanced mitotic activity, nuclear pleomorphism, conspicuous nucleoli, and necrosis ^23^. In such cases, wide local excision with clear margins is indicated to prevent local recurrence.

### Lymphomas and Plasmacytoma

Primary breast lymphomas are rare, with an incidence of 0.04-1% of malignant breast neoplasms ^24^. Among the subtypes, various studies have elucidated that the most common occurrence is of diffuse large B cell lymphoma, which occurs in more than 50% of primary breast lymphomas ^25,26^. Each subtype is known to have a distinct epidemiology, treatment modality, and prognostication.

Often presenting as a rapidly growing lump and radiologically diagnosed usually as carcinoma, these entities require histomorphological evaluation and a wide panel of IHC for their sub-categorization. The treatment of primary breast lymphomas remains controversial; as there is no consensus on the best approach. However, certain guidelines have been established, and they may be based on a combination of surgery, chemoradiation, and immunotherapy ^26^.

Another rarity is breast plasmacytomas, which have an incidence of approximately 1.5% of all the plasmacytomas that occur. Nearly 15% of these are classified as primary, while the others are secondary to multiple myeloma ^27^. These are characterized by the proliferation of neoplastic plasma cells, and due to their rarity, they might be disregarded as chronic inflammation.

The recurrence rate of breast plasmacytomas is around 25%, warranting the need for chemotherapy or radiotherapy ^28^. Due to the rarity of the presentation, standard treatment guidelines are not available, but wide local excision with or without radiation is the treatment mainstay.

This study corroborates and expands upon global data on rare breast neoplasms while providing regional insights. The high incidence of triple-negative receptor profiles in adenoid cystic carcinoma aligns with findings by Ghabach et al. and Kim et al., highlighting its distinct clinical behavior and favorable prognosis. Similarly, the aggressive progression of neuroendocrine carcinomas observed here supports global trends reported by Bogina et al., emphasizing the need for tailored management strategies. Unique to this study is the relatively higher proportion of hematolymphoid neoplasms and myofibroblastomas, potentially reflecting regional genetic or diagnostic differences. Furthermore, the detailed characterization of granular cell tumors and hamartomas adds valuable data to underrepresented entities in the literature.

## Conclusion

The pathologist’s mainstay in the diagnosis of rarer entities is vast, as breast neoplasms, due to their heterogeneous nature, pose a hassle in routine diagnosis. Moreover, the varying types of breast neoplasms described in the blue book demonstrate a contrasting prognostic and predictive behavior. Hence, a more comprehensive study of the clinical and genomic aspects of unusual histological subtypes of breast neoplasm is required to provide a more personalized treatment decision.
